# A survey and cause analysis of community resilience in a Chinese city from the perspective of nursing

**DOI:** 10.1186/s12889-021-12331-1

**Published:** 2022-01-15

**Authors:** N. Jiang, L. H. Ma, J. X. Cheng, X. L. Jiang

**Affiliations:** 1 West China School of Nursing/West China Hospital, Sichuan University, Chengdu, 610044 Sichuan China; 2 Institute for Disaster Management and Reconstruction, Sichuan University, Chengdu, 610207 Sichuan China

**Keywords:** Nursing, Earthquake disaster, Urban community resilience, Evaluation system, Cause analysis

## Abstract

**Background:**

Community resilience, which fully reflects the ability of communities to resist, absorb, recover or adapt to disasters, has attracted international attention. Nurses are an important force in disaster prevention, relief and postdisaster reconstruction. This study aims to test the current level of community resilience in Dujiangyan city, which was seriously damaged by the Wenchuan earthquake, and analyze the causes.

**Methods:**

Community data from 952 residents, 574 families, 5 health care institutions and 12 communities in Dujiangyan city were collected by using stratified, cluster, map and systematic sampling methods. A new community resilience evaluation system from the perspective of nursing was used to test individual, family, health care and environmental resilience.

**Results:**

In Dujiangyan city, average scores were obtained for community resilience (3.93 ± 0.12), individual resilience (4.07 ± 0.64), family resilience (4.07 ± 0.6), health care resilience (3.84 ± 0.33) and community environment resilience (3.69 ± 0.46).

**Conclusions:**

The urban communities in Dujiangyan city had acceptable resilience, with good family and individual resilience and medium health care and community environment resilience, but environmental resilience had the lowest score. Because conditions and resilience levels varied among the communities, targeted measures should be taken to improve resilience based on population characteristics, management, professional organizations, hardware and software facilities.

**Supplementary Information:**

The online version contains supplementary material available at 10.1186/s12889-021-12331-1.

## Background

Earthquakes are a destructive natural disaster. Strong earthquakes can cause high casualties and major economic losses, thereby seriously restricting the sustainable development of society. With the acceleration of urbanization, cities are becoming more vulnerable to earthquake disasters [1]. The community is the basic constituent unit of a city. When a disaster occurs, the community is the first to be affected by the disaster, but the community can also carry out initial disaster relief activities. Resilience generally refers to the ability of a system to recover to its original state after being disturbed [2], and community resilience fully reflects the ability of a community to resist, absorb, recover or adapt after a disaster. In this study, community resilience is defined as the ability of communities exposed to earthquake disasters to promptly absorb, resist, adapt to and recover from these disasters to reduce the losses experienced by individuals, families and the environment. Disaster tolerance, response and recovery are the basic characteristics of community resilience.

Evaluation methods are an important part of community resilience research. Researchers have developed many community resilience assessment tools that adopt different perspectives. The community disaster resilience index developed by Mayunga includes five dimensions: social capital, economic capital, population capital, physical capital and natural capital. The scores of each dimension are calculated, and the mean value is obtained to evaluate community resilience [3]. Cutter et al. designed the baseline resilience index for communities, which includes the following five dimensions: society, economy, organization, infrastructure and community capital. By standardizing the data and adding up the dimensions, a region’s resilience score is obtained [4]. Shaw et al. designed the Climate Disaster Resilience Index, which includes physical, social, economic, institutional and natural dimensions and calculates the resilience by using a weighted average [5]. Ainuddin et al. constructed a community resilience indicator framework that includes 4 primary indicators—social, economic, institutional and physical resilience—and 15 secondary indicators [6]. Asad Asadzade et al. constructed the seismic resilience index model based on the network analytic hierarchy process; this model includes 8 first-level indexes and 26 s-level indexes and obtains the final resilience score by using the linear weighting method [7]. Mishra et al. designed the postearthquake recovery framework, which includes 4 primary indicators—psychological recovery, infrastructure recovery, economic recovery and social recovery—and 13 secondary indicators [8].

As an important force in community disaster prevention and mitigation, nursing staff not only directly participate in on-site rescue but also play an irreplaceable role in predisaster prevention and postdisaster reconstruction [9]. Most of the current evaluation tools are based on social management or engineering technology and collect mainly objective data. Nursing focuses on people, the environment and health; therefore, current research tools are not available for nursing. Based on this gap, we constructed a system for evaluating community resilience from the perspective of nursing under the core concepts of nursing; we then combined subjective and objective data to comprehensively reflect community resilience. First, we summarize the assessment focus of 52 community resilience evaluation tools published in the past 12 years. Then, the framework of community resilience was formed by using expert interviews and considering previous assessments. Finally, by using a literature review and the Delphi method, we constructed a new community resilience evaluation system based on earthquake disasters from the perspective of nursing (CRES-EN). The CRES-EN consists of four questionnaires: an individual resilience questionnaire, a family resilience questionnaire, a health care resilience questionnaire and a community environmental resilience questionnaire. The calculation formula is Y = 0.343X_I_ + 0.183X_F_ + 0.25X_H_ + 0.224X_E_.

Dujiangyan city, which belongs to Sichuan Province, is located on the northwest edge of the Chengdu Plain and covers an area of 1208 km^2^; the city has 5 subdistrict offices, 14 towns, and 1 development zone. The resident population of the city is nearly 700,000, and the urbanization rate is 60.2%. On May 12, 2008, Wenchuan was hit by an 8.0-magnitude earthquake. The downtown area of Dujiangyan city was only about 20 km from the epicenter. The earthquake seriously damaged the downtown area of Dujiangyan city, which was listed by the Chinese government as one of the areas worst hit by the Wenchuan earthquake.

As an area that experiences strong earthquakes, Dujiangyan city has experience and advantages in building community resilience. The city is located on a large fault line and thus is at risk of experiencing another great earthquake. This study selected urban communities in Dujiangyan city for the resilience assessment. By collecting data, we can understand the current situation of urban community resilience in Dujiangyan city.

## Methods

We conducted the investigation in Dujiangyan city from July to November 2019 by the following methods.

### Subjects and inclusion criteria

#### Residents

Inclusion criteria: Residents who 1) had lived in the community for more than half a year, 2) were at least 15 years old, 3) could complete questionnaires independently or with the help of investigators, and 4) provided informed consent.

#### Communities

Inclusion criteria: An urban community consisting mainly of urban residents. 

#### Community health care institutions

Inclusion criteria: A registered community health care center or community hospital. 

### Sample size

#### Community

Dujiangyan city has 5 subdistrict offices with 45 urban communities. We used a stratified sampling method to select 12 communities according to the proportion of 25%.

#### Community health care institutions

Dujiangyan city has 5 community health centers, all of which were included in the study.

#### Households

Map sampling and systematic sampling methods were adopted to identify households. The twelve sampling communities contain approximately 38,500 households; with a proportion of 15‰, the sample size for households was approximately 577 (38,500 households × 15‰).

#### Residents

Cluster sampling was used to select family members from the sampling households. Based on each household having 2.6 family members over the age of 15 [10], this study needed approximately 1500 residents (577 households × 2.6).

### Survey tool

The survey tool consists of two parts. The first part captures basic community information, such as the community’s name, geographical location, establishment year, population and community area. The second part is the CRES-EN, which was developed for this study (Supplementary File 1 and Supplementary File 2). It includes the individual resilience questionnaire (4 dimensions and 17 items, with content validity 0.94 and Cronbach’s α 0.944), family resilience questionnaire (4 dimensions and 14 items with content validity 0.93 and Cronbach’s α 0.894), health care resilience questionnaire (4 dimensions and 17 items with content validity 0.95 and Cronbach’s α 0.906) and environment resilience questionnaire (3 dimensions and 23 items with content validity 0.93 and Cronbach’s α 0.875).

### Data collection

The family resilience questionnaire was completed by a family member who was familiar with the family situation, and then each family member completed an individual resilience questionnaire. Hospital managers and medical staff completed the health care resilience questionnaire. The community environmental resilience questionnaire was completed by government staff responsible for community matters. Our team members also interviewed some community office workers and residents to gain insight into the causes of the current state of community resilience. The questions included mainly what the community, medical institutions, families or individuals had done to prepare and respond to disasters, exploring what the advantages were of these efforts in promoting resilience and what problems still exist. The data reported here are part of a much larger mixed-methods study that also included 47 qualitative interviews. These data will be reported in another article.

### Statistics

SPSS Statistics for Windows, Version 19.0 (SPSS Inc.; Armonk, NY: IBM Corp.) was used for the data analysis. The frequency/component ratio was used to describe the count data, while the measurement data were described with the item mean ± SD. According to the above calculation formula for community resilience, the item mean was multiplied by the corresponding weight, and the sum was the current level of community resilience, which was expressed as the mean ± SD.

## Results

### Basic information on subjects

#### Residents

A total of 1048 questionnaires were sent to residents, and 952 were returned, resulting in a response rate of 90.84%. In residents, male vs. female was 40.34% vs. 59.66%. The majority of the residents surveyed were between 30 and 60 years old (71.85%), and people with a high school education or above accounted for 57.77%. A total of 43.07% of the residents had jobs, and the annual income of most residents was less than 40,000 yuan. (Table 1).

**Table 1:** Basic information of residents in Dujiangyan city (*n* = 952)

Content	Category	Number of residents (n)	Constituent ratio (%)
Sex	Male	384	40.34
	Female	568	59.66
Age	Under 18	25	2.63
	18~	122	12.82
	30~	184	19.33
	40~	165	17.33
	50~	199	20.9
	60~	136	14.29
	70~	121	12.71
Education	Primary school and below	159	16.7
	Junior high school	243	25.53
	Senior high school	247	25.95
	Junior college and above	303	31.83
Employment	Yes	410	43.07
	No	185	19.43
	Retirement	298	31.3
	Student	59	6.2
Annual disposable income (714 residents responded)	Less than 20,000 RMB	90	12.61
	20,000~	296	41.46
	40,000~	181	25.35
	60,000~	69	9.66
	80,000~	78	10.92

#### Families

A total of 577 questionnaires were sent to residents, and 574 were returned, resulting in a response rate of 99.48%. In 574 families, the proportions of the number of family members were 1–2 (33.91%), 3–4 (47.01%), 5–6 (17.92%) and up to 7 (1.16%). A total of 89.51% of families had their own properties in the community, and only 10.49% of families had no properties. (Table 2).

**Table 2:** Basic information of families in Dujiangyan city (*n* = 574)

Content		Family numbers (n)	Constituent ratio (%)
Subordinate to subdistricts	a subdistrict	169	29.44
	b subdistrict	192	33.45
	c subdistrict	49	8.54
	d subdistrict	48	8.36
	e subdistrict	116	20.21
Family members	1–2	194	33.91
	3–4	270	47.01
	5–6	103	17.92
	7~	7	1.16
Property ownership	Owner-occupied property	514	89.51
	Non-owner-occupied property	60	10.49

#### Community health care institutions

A total of 5 questionnaires were sent to community health care institutions, and 5 were returned, resulting in a response rate of 100%. All 5 community health care institutions were built under the standard of Level I (in China, hospitals are built according to three grades, level 1 is the basic level and level 3 is the highest level). The average number of medical staff was 35.4, of which more than three-quarters had college degrees or above, and most of them had junior professional titles. (Table 3).

**Table 3:** Basic information of community health care institutions in Dujiangyan city (*n* = 5)

Code	Level	Medical staff							Bed Number
		Number	Education background			Professional title			
			Bachelor degree or above	College degree	Technical school graduate	Senior	Medium	Junior	
A	1	32	9	21	2	3	6	23	60
B	1	36	9	16	11	0	8	28	90
C	1	34	5	18	11	1	5	28	50
D	1	40	18	19	3	0	11	29	60
E	1	35	12	18	5	2	7	26	65

#### Communities

A total of 12 questionnaires were sent to urban communities in Dujiangyan city, and 12 were returned, resulting in a response rate of 100%. The average time of community establishment was 12.58 years, and the average area of the community was 0.85 km^2^. Each community had an average of approximately 7000 residents, and the number of residents with urban household registration accounted for 89.98%. (Table 4).

**Table 4:** Basic information of communities in Dujiangyan city (*n* = 12)

Code	Established year	Area (sq.km.)	Residents number (n)	Male-female ratio	Urban residents (%)
I	2011	0.65	5379	1:1.25	100
II	2010	1.5	6969	1:1.01	100
III	2010	1.13	11,500	1:1.06	100
IV	2010	0.7	1085	1:0.67	66.7
V	2002	0.11	927	None	100
VI	2007	0.4	3824	1:0.83	90
VII	1994	0.75	2573	1:1.04	75
VIII	2004	1.15	12,563	None	55
IX	2002	0.9	16,369	1:1.01	100
X	2009	0.5	4200	1:0.97	98
XI	2007	1.2	11,560	None	95
XII	2011	1.2	8135	1:1	100

### Scores

#### Average score of community resilience in Dujiangyan city

In Dujiangyan city, the resilience level of urban communities was acceptable, with good family and individual resilience, medium health care and environment resilience, but the community environment resilience received the lowest score.

The average score for community resilience in Dujiangyan city was 3.93 ± 0.12. The four subscales had scores of 4.07 ± 0.64 for individual resilience, 4.07 ± 0.6 for family resilience, 3.84 ± 0.33 for health care resilience and 3.69 ± 0.46 for community environment resilience. For individual resilience, the health status dimension had the highest score (4.39 ± 0.67), and the earthquake disaster response ability dimension had the lowest score (3.5 ± 0.94). In family resilience, the family relationship dimension scored the highest (4.33 ± 0.7), and crisis response scored the lowest (3.69 ± 0.81). For health care resilience, the dimensions with the highest and lowest scores were medical resource (4.67 ± 0.34) and medical staff disaster management capacity (3.16 ± 0.44), respectively. The score of disaster preparedness and response management (4.03 ± 0.62) in environmental resilience was higher than that of economic capital (2.76 ± 0.63). (Table 5).

**Table 5:** Average score of community resilience in Dujiangyan city (*n* = 12)

Questionnaires	Content				Score
Individual resilience	Health status	Psychological resilience	Social adaptation	Disaster response capacity	
	4.39 ± 0.67	4.2 ± 0.71	4.16 ± 0.68	3.5 ± 0.94	4.07 ± 0.64
Family resilience	Family faith	Family relationship	External support	Crisis response	
	4.21 ± 0.84	4.33 ± 0.7	4.14 ± 0.72	3.69 ± 0.81	4.07 ± 0.6
Health care resilience	Medical resource	Hospital emergency management	Overload capacity	Medical staff disaster management capacity	
	4.67 ± 0.34	3.53 ± 0.69	3.67 ± 0.45	3.16 ± 0.44	3.84 ± 0.33
Community environment resilience	Economic capital	Disaster preparedness and response management	Infrastructure		
	2.76 ± 0.63	4.03 ± 0.62	3.96 ± 0.74		3.69 ± 0.46
Total score		3.93 ± 0.12			

#### Resilience scores of the 12 communities

Concerning the community resilience scores, community II (4.12) had the highest, while community X (3.74) had the lowest. Concerning the individual resilience scores, community I (4.45) had the highest, while community VI (3.78) had the lowest. Concerning the family resilience scores, community I (4.51) had the highest, while community VI (3.75) had the lowest. Concerning the health care resilience scores, the community E healthcare center (4.33) had the highest, while the community A health care center (3.43) had the lowest. Concerning the community environmental resilience scores community II (4.39) had the highest, while community IV (3.05) had the lowest (Table 6).

**Table 6:** Resilience scores of communities in Dujiangyan city (*n* = 12)

Code	Individual resilience	Family resilience	Health care resilience	Community environment resilience	Total score
I	4.45 ± 0.54	4.51 ± 0.43	3.93	3.14	4.04
II	3.97 ± 0.71	4.31 ± 0.5	3.93	4.39	4.12
III	4 ± 0.62	4.13 ± 0.46	3.93	4.01	4.01
IV	3.94 ± 0.9	4.24 ± 0.55	4.33	3.05	3.89
V	3.9 ± 0.39	3.97 ± 0.66	4.33	4.31	4.11
VI	3.78 ± 0.63	3.75 ± 0.67	3.69	3.88	3.77
VII	4.14 ± 0.68	4.1 ± 0.7	3.69	4.02	3.99
VIII	4.15 ± 0.63	4.11 ± 0.66	3.69	3.84	3.96
IX	3.94 ± 0.56	3.83 ± 0.55	4.33	3.63	3.95
X	4.07 ± 0.65	4.11 ± 0.64	3.43	3.28	3.74
XI	4.22 ± 0.67	3.85 ± 0.69	3.43	3.49	3.79
XII	4.17 ± 0.7	4.24 ± 0.58	3.83	3.17	3.87

#### Subdistrict scores

The resilience scores of the 5 subdistricts under the jurisdiction of Dujiangyan city were as follows: a subdistrict 4.06 ± 0.06, b subdistrict 3.96 ± 0.01, c subdistrict 3.94 ± 0.14, d subdistrict 3.87, and e subdistrict 3.77 ± 0.04 (Table 7).

**Table 7:** Resilience scores of the 5 subdistricts in Dujiangyan city (*n* = 5)

Code	Individual resilience	Family resilience	Health care resilience	Community environment resilience	Total score
a	4.14 ± 0.27	4.32 ± 0.19	3.93	3.85 ± 0.64	4.06 ± 0.06
b	4.05 ± 0.15	3.97 ± 0.2	4.01	3.74 ± 0.15	3.96 ± 0.01
c	3.94 ± 0.15	4.02 ± 0.21	4.01	3.82 ± 0.54	3.94 ± 0.14
d	4.17 ± 0.7	4.24 ± 0.58	3.83	3.17	3.87
e	4.15 ± 0.11	3.98 ± 0.18	3.43	3.39 ± 0.15	3.77 ± 0.04

#### Resilience map of Dujiangyan city

**Fig. 1:**
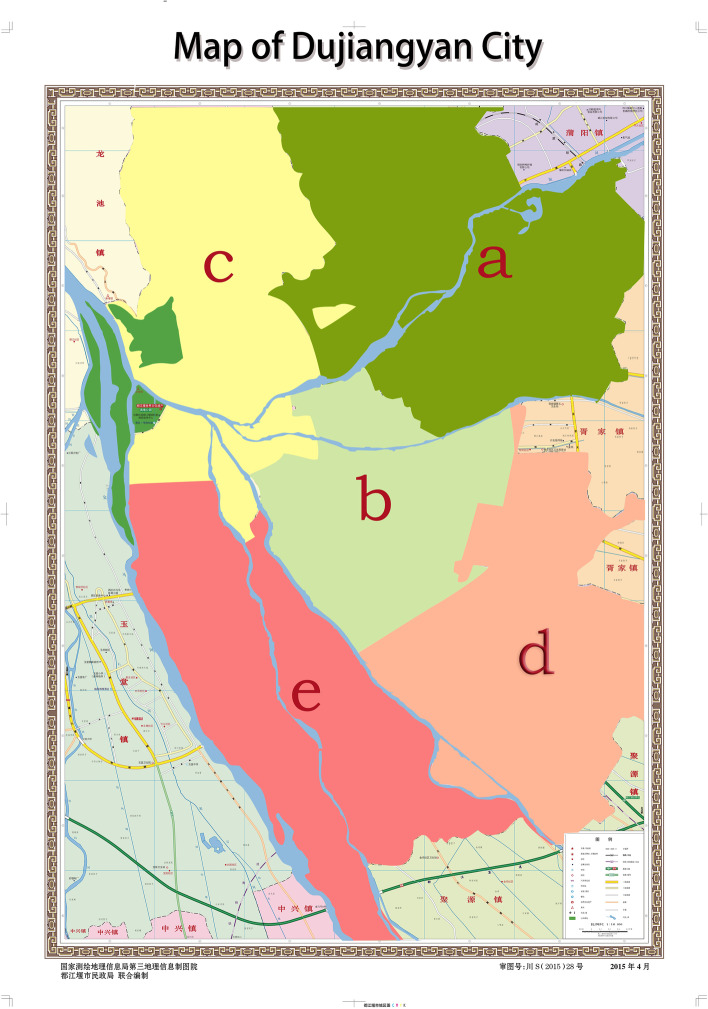
Resilience map of the urban area in Dujiangyan city. The Dujiangyan city map was jointly produced by the Ministry of Natural Resources of China and the Dujiangyan municipal government which is freely available on public websites and has been orally approved by Dujiangyan government staff. Available website: https://www.51wendang.com/doc/2ed48ad0b522131e18e2c210

To draw the resilience map, the map of the urban area of Dujiangyan city is colored according to the resilience scores of the 5 subdistricts. The higher the score is, the better the resilience. The subdistricts with the highest, second highest, middle, second lowest, and lowest scores were marked in dark green, light green, yellow, light red, and dark red, respectively (Fig. 1).

## Discussion

Most studies have focused on public administration or infrastructure to evaluate community resilience [4–6, 8], but ignored the impact of the subjective initiative of community residents and families. The CRES-EN comprehensively evaluates community resilience from multiple perspectives. We divided the community system into four subsystems, namely, individual, family, health care and community environment, and these four subsystems are closely linked to the outcomes of earthquake disasters. Nursing focuses on people and their environment and health, and residents are an important part of a community; thus, their subjective initiative is crucial for building resilience in the community. Therefore, the evaluation system developed in this study took individual resilience as the core, emphasized mutual support through the family and health care system with regard to environment resilience, and conducted a comprehensive evaluation by combining subjective and objective indicators. In particular, the study evaluated individual resilience, family resilience and health care system resilience, which are the key areas in which nursing professionals can intervene to improve resilience in urban communities.

### Individual resilience

In the face of disaster events, a resilient individual should have a healthy body, a tough character, good social adaptability and a strong disaster response ability. Most current studies take objective data, such as the incidence of chronic diseases, population composition, income and psychological status, as indicators of community population resilience. However, these data may be updated slowly and fail to reflect the actual problems [3, 6, 7, 11, 12]. Community residents know their individual condition best. In this study, existing health data on the community and residents’ self-evaluation are combined to comprehensively describe the individual resilience of community residents to better reflect the actual level of resilience. Among the types of individual resilience investigated in this study, health status, psychological resilience and social adaptability were good, but earthquake disaster response ability was poor. Generally, people who have experienced disaster events can improve their psychological resilience and social adaptability to a certain extent, which is consistent with existing studies [13–15].

The state of physical health is mainly related to the extent of the disaster, the level of local economic development, postdisaster recovery and the characteristics of the population [16–18]. In this study, due to the damage of the Wenchuan earthquake to the urban area of Dujiangyan city, the government demarcated different postdisaster reconstruction communities for housing resettlement according to the characteristics of local residents. To encourage the integration of residents, many communities have organized appealing activities, such as kite festivals, dam feasts, and festival parties. These activities not only enrich daily life but also improve individual resilience in terms of physiology, psychology and adaptability and can therefore serve as a reference for interventions aimed at improving individual resilience.

Although Ye believed that residents’ self-rescue and mutual rescue abilities were an indispensable part of emergency rescue [15], in this study, on-site rescue ability had the lowest score. This ability refers mainly to the rescue of buried people, the treatment of wounds, freehand cardiopulmonary resuscitation and basic handling methods. Many residents said that these skills were too professional to be well mastered. This statement suggests that we should encourage and support nursing professionals in improving the on-site rescue ability of community residents by teaching rescue knowledge and conducting skills training that will aid in preparation for an earthquake disaster.

### Family resilience

Family relationships are the cornerstone of family resilience, and good family cohesion enables family members to work better together in response to crises [19]. A positive attitude with regard to overcoming difficulties is the core of family resilience [20]. In this study, family relationships and family beliefs scored high. Many residents had experienced the earthquake or had personally participated in the process of postdisaster reconstruction, so their intentions to provide mutual support and encourage optimistic attitudes were relatively strong.

In the later interview, we found that residents with higher education generally had more stable families and careers than residents with less education, and their higher knowledge level and greater experience helped them to be independent and able to accept new challenges with a more tenacious attitude; this could allow them to more actively respond in a crisis [21]. As a supportive resource, social support could help families flexibly cope with various life events [22]. Professional community support is an important aspect of family resilience [20]. However, in this study, residents’ recognition of the support provided by the community was not high. On the one hand, this result may be due to cultural background, as Chinese people have always advocated self-reliance; they are embarrassed to solicit help from government agencies and try their best to solve their own affairs. On the other hand, some communities cannot put their daily work into practice. This suggests that community workers should strengthen contact with residents and put in place comprehensive community strategies. The family crisis response dimension had the lowest score. Lindsey et al. believed that the economic foundation is an important source of support for family resilience [19]. Earthquakes can cause damage to homes, property and so on. Additionally, following an earthquake, the battered local economy will offer fewer opportunities for residents to obtain jobs [23, 24]. Households with reduced incomes did not have extra money to spend on emergency food or medicine stocks, so disaster response capacity was lower. In a later investigation, we also found that many families were not very active in dealing with earthquake disasters and were not confident in their own abilities related to disaster prevention and mitigation.

### Health care resilience

In terms of health care resilience, the medical resource dimension had the highest score. After the Wenchuan earthquake, medical institutions in Dujiangyan city were severely damaged. Most of the community hospitals were built with assistance from Shanghai city, and because of Shanghai city’s resource advantages, the software and hardware resources of the community hospitals were good. The medical consortium policy promoted by the state has been widely implemented in China. All community hospitals had tertiary or secondary hospitals in the same region as consortium hospitals or two-way referral hospitals that could provide support or assistance in terms of referrals, technical support, staff training and so on. In terms of emergency resources, most hospital emergency supplies were distributed through cooperation agreements with suppliers. Community hospitals, as primary medical institutions, mainly play a role in the transitional stage before external relief arrives; community hospitals have limited funds, so having a large reserve of emergency resources is impractical for these institutions. Therefore, having a small amount of material reserves and cooperation agreements with suppliers is a practical approach.

Medical staff disaster management capacity had the lowest score among four dimensions. Ranked in descending order by score, these were communication ability, on-site emergency handling ability, disaster mitigation and preparedness ability, disaster assessment ability, and postdisaster disposal ability. This ranking is consistent with the results of Yang Yana [25]. Community health care workers have to engage with a variety of community residents and often go to residents’ homes to provide health education, so health care workers must have good communication skills. Most of the training in hospitals involved on-site emergency treatment capacity, such as cardiopulmonary resuscitation, hemostasis, and dressing, but there was little training related to disaster reduction and preparedness, disaster assessment and postdisaster disposal methods. This suggests that community medical institutions should not limit training to on-site first aid but should offer training related to disaster assessment and management and postdisaster reconstruction.

### Community environment resilience

The dimensions of community environment resilience included three aspects: disaster preparedness and response management, infrastructure, and economic capital. Ainuddin et al. believed that a strong economy can not only enable communities to recover quickly from the impact of disasters but also reduce the risk of future disasters [6]. Dujiangyan city was hit hard by the Wenchuan earthquake in 2008, resulting in a slow economic recovery. However, the local government attaches great importance to earthquake emergency management, so the resilience of the disaster preparedness management system was good, and this was mainly reflected in the emergency command team, emergency planning, material reserves, and residents’ participation.

Infrastructure is an important aspect of community environmental resilience. Good infrastructure can withstand the consequences of earthquake disasters, keep basic functions intact, provide effective guarantees for reducing the number of residents’ lives lost, reduce property losses, and promote postdisaster recovery [11, 26, 27]. In this study, the infrastructure resilience was medium, but the scores for water supply, power supply and housing were low. A field survey showed that the housing quality of communities is uneven. On the one hand, half of the communities were rebuilt after the Wenchuan earthquake, and seismic performance was emphasized at the beginning of reconstruction. Therefore, the seismic resilience of the water supply, power supply and housing in these communities were good. On the other hand, due to the limitations of the local economy, other houses that were not seriously damaged were reinforced rather than reconstructed. This resulted in severe aging of the water and power supply facilities, and the seismic performance of these buildings was poor. In addition, some managers did not pay enough attention to infrastructure resilience, so the scores were low, which was consistent with existing research [28]. This suggests that the economy, management and infrastructure mutually reinforce and influence each other [29, 30]. A reduction in the resilience of one of these aspects will affect the other two, thereby reducing the overall environmental resilience of the community. Managers should pay attention to these three aspects and endeavor to balance their development to achieve an overall improvement in community environmental resilience.

## Conclusion

The evaluation system used in this study took individual resilience as the core and emphasized mutual support through the family resilience, health care resilience, and environment resilience, which could assess community resilience from a more holistic perspective. The resilience of urban communities in Dujiangyan city was medium, with family and individual resilience being high and health care and community environment resilience being medium, but community environment resilience had the lowest score. As the specific situation and resilience levels varied among communities, targeted measures should be taken to improve community resilience according to the actual situation while considering factors such as population characteristics, hardware and software facilities, and areas of strength and weakness in the resilience measurements. The samples of this study were mainly from communities in China, so the representativeness of the study was limited, and the extrapolation of the results might be affected. We recommend that large sample studies be carried out in urban communities with different national and cultural backgrounds to support our findings. Compared with urban communities, rural communities have their own characteristics and may be less resilient to disasters. It was suggested to further enrich the evaluation system of the community resilience for earthquake disasters and carry out the assessment of the resilience of rural communities to provide a scientific basis for the improvement of the overall community resilience.

## Supplementary Information

**Additional file 1.** Community Resilience Evaluation System based on Earthquake disasters from the perspective of Nursing---English Version.

**Additional file 2.** Weights of three levels in the Community Resilience Evaluation System.

## Data Availability

The datasets used and/or analysed during the current study are available from the corresponding author on reasonable request.

## Abbreviation

CRES-EN: Community Resilience Evaluation System based on Earthquake disasters from the perspective of Nursing
