# Supplementary material for: A survey and cause analysis of community resilience in a Chinese city from the perspective of nursing
Source: BMC Public Health. 2022 Jan 15;22:2. doi: 10.1186/s12889-021-12331-1 (PMC8760825; doi:10.1186/s12889-021-12331-1)
Supplement: Supplementary file 1 — Additional file 1. Community Resilience Evaluation System based on Earthquake disasters from the perspective of Nursing---English Version. [file 12889_2021_12331_MOESM1_ESM.docx]

**Community Resilience Evaluation System based on Earthquake disasters from the perspective of Nursing (CRES-EN)**

**English Version**

**1. Basic Information of community**

Name of the community:

Community location:

Establishment time:

Community area:

Community population

Number of households in the community:

Number of community residents:

**2. CRES-EN**

**Individual resilience**

| First-level | Second-level | Third-level | Standard score |
| --- | --- | --- | --- |
| Individual resilience | Health status | I am generally in good health. | 5 --- Extremely match  4 --- Good match  3 --- Average match  2 --- Poor match  1 --- Very poor match |
|  |  | I can move freely and easily. | 5 --- Extremely match  4 --- Good match  3 --- Average match  2 --- Poor match  1 --- Very poor match |
|  |  | I can think clearly and communicate with others. | 5 --- Extremely match  4 --- Good match  3 --- Average match  2 --- Poor match  1 --- Very poor match |
|  | Mental resilience | I believe I can control my emotions when an earthquake strikes. | 5 --- Extremely match  4 --- Good match  3 --- Average match  2 --- Poor match  1 --- Very poor match |
|  |  | I always have a positive view when suffering difficulties. | 5 --- Extremely match  4 --- Good match  3 --- Average match  2 --- Poor match  1 --- Very poor match |
|  |  | I do not give up easily when suffering difficulties. | 5 --- Extremely match  4 --- Good match  3 --- Average match  2 --- Poor match  1 --- Very poor match |
|  |  | I can recover from setbacks quickly. | 5 --- Extremely match  4 --- Good match  3 --- Average match  2 --- Poor match  1 --- Very poor match |
|  |  | I believe that difficulties make me stronger. | 5 --- Extremely match  4 --- Good match  3 --- Average match  2 --- Poor match  1 --- Very poor match |
|  | Social adaptation | I can play my roles in daily life well, such as studying hard as a student, doing my job well as a worker, taking care of my families as a parent, etc. | 5 --- Extremely match  4 --- Good match  3 --- Average match  2 --- Poor match  1 --- Very poor match |
|  |  | I can cope with problems well. | 5 --- Extremely match  4 --- Good match  3 --- Average match  2 --- Poor match  1 --- Very poor match |
|  |  | I can adapt quickly when the environment changes. | 5 --- Extremely match  4 --- Good match  3 --- Average match  2 --- Poor match  1 --- Very poor match |
|  |  | I can get along with others well. | 5 --- Extremely match  4 --- Good match  3 --- Average match  2 --- Poor match  1 --- Very poor match |
|  |  | I am good at finding and using social resource (staff, funds, supplies, skills, social relations and so on). | 5 --- Extremely match  4 --- Good match  3 --- Average match  2 --- Poor match  1 --- Very poor match |
|  | Disaster response capacity | I have basic ability of earthquake disaster assessment .  Index illumination: I am aware of potential earthquake threats, can perceive earthquake warning signs, and assess the level of an earthquake and the risk of secondary disaster preliminarily. | 5 --- Extremely match  4 --- Good match  3 --- Average match  2 --- Poor match  1 --- Very poor match |
|  |  | I know how to escape or avoid danger indoors and outdoors.  Index illumination: I know how to escape or avoid danger indoors and outdoors, and self-rescue when trapped. | 5 --- Extremely match  4 --- Good match  3 --- Average match  2 --- Poor match  1 --- Very poor match |
|  |  | I have basic survival skills after an earthquake.  Index illumination: I have skills including getting water, food, and fuel and keeping water and food hygeian. | 5 --- Extremely match  4 --- Good match  3 --- Average match  2 --- Poor match  1 --- Very poor match |
|  |  | I can administer first aid.  Index illumination: I know the principles of searching for and rescuing those who are buried and have preliminary skills of first aid including hemostasis, bandages, CPR and carrying the wounded. | 5 --- Extremely match  4 --- Good match  3 --- Average match  2 --- Poor match  1 --- Very poor match |

**Family resilience**

| First-level | Second-level | Third-level | Standard score |
| --- | --- | --- | --- |
| Family resilience | Family belief | No matter how big the difficulties are, my families always face them bravely. | 5 --- Extremely match  4 --- Good match  3 --- Average match  2 --- Poor match  1 --- Very poor match |
|  |  | We know that earthquakes are inevitable, but we believe that with the efforts of the whole families, the damage can be minimized. | 5 --- Extremely match  4 --- Good match  3 --- Average match  2 --- Poor match  1 --- Very poor match |
|  | Family relationship | My families are very united, and we can support each other. | 5 --- Extremely match  4 --- Good match  3 --- Average match  2 --- Poor match  1 --- Very poor match |
|  |  | Communication between family members is frequent and smooth. | 5 --- Extremely match  4 --- Good match  3 --- Average match  2 --- Poor match  1 --- Very poor match |
|  |  | When disagreements arise between family members, we negotiate together to reach a consensus. | 5 --- Extremely match  4 --- Good match  3 --- Average match  2 --- Poor match  1 --- Very poor match |
|  |  | Relationship of the families are close, and we can  understand and tolerate each other. | 5 --- Extremely match  4 --- Good match  3 --- Average match  2 --- Poor match  1 --- Very poor match |
|  | External support | We have close friends who can help us when we need help. | 5 --- Extremely match  4 --- Good match  3 --- Average match  2 --- Poor match  1 --- Very poor match |
|  |  | We have a harmonious relationship with our neighbors  and can help each other in case of an earthquake. | 5 --- Extremely match  4 --- Good match  3 --- Average match  2 --- Poor match  1 --- Very poor match |
|  |  | When we are in trouble, our relatives will come to help us. | 5 --- Extremely match  4 --- Good match  3 --- Average match  2 --- Poor match  1 --- Very poor match |
|  |  | Community agencies (such as neighborhood committees, police stations, community hospitals, etc.) will help us when we needed. | 5 --- Extremely match  4 --- Good match  3 --- Average match  2 --- Poor match  1 --- Very poor match |
|  | Crisis response | We have an earthquake emergency kit in our house.  Index illumination:  ● Emergency food, including food and bottled water that can be stored for at least 3 days/person.  ● Emergency medical kit, including disinfectant, gauze, hemostatic, painkiller, antidiarrheal, antipyretic, etc.  ● Emergency tools kit, including flashlights, blankets, radios, safety ropes, raincoats, whistles, etc. | 5 --- All three types of emergency supplies are available and complete  4 --- All three types of emergency supplies are available but not complete  3 --- Only have two types  2 --- Only have one type  1 --- No emergency supplies |
|  |  | My family is financially sound and able to cope with life's difficulties. | 5 --- Extremely match  4 --- Good match  3 --- Average match  2 --- Poor match  1 --- Very poor match |
|  |  | If an earthquake occurs, we can work together to deal with the disaster. | 5 --- Extremely match  4 --- Good match  3 --- Average match  2 --- Poor match  1 --- Very poor match |
|  |  | When existing methods don't work, we try to find new ways to get out of trouble. | 5 --- Extremely match  4 --- Good match  3 --- Average match  2 --- Poor match  1 --- Very poor match |

**Health care resilience**

| First-level | Second-level | Third-level | Standard score |
| --- | --- | --- | --- |
| Health care resiience | Medical resource | The ratio of the number of community health centres to the community population meets national standard.  Index illumination: A community hospital or community health service center serves 30,000 to 100,000 residents. Communities with a population larger than 100,000 should set up more than one community health centers accordingly. | 5 --- Extremely match  1 --- Very poor match |
|  |  | The number of health care workers per thousand population in the community.  Index illumination: The number of health care workers per 1,000 people in a community should reach the standard of 0.58. | 5 --- Above 0.77  4 --- 0.65-0.77  3 --- 0.52-0.64  2 --- 0.39-0.51  1 --- Under 0.39 |
|  |  | There are cooperative superior medical institutions as assistance, support or collaboration units.  Index illumination: The community medical institution has a cooperative superior hospital which can carry out patient referral, diagnosis and treatment guidance, staff training, etc. | 5 --- Extremely match  4 --- Good match  3 --- Average match  2 --- Poor match  1 --- Very poor match |
|  | Hospital emergency management | The hospital has an earthquake emergency command team.  Index illumination: Team members should include hospital leaders, doctors, nurses, pharmacists, imaging, laboratory and logistics leaders, and an appointed person responsible for coordination within and outside the hospital. | 5 --- The team members are complete, and appointing a person responsible for coordination  4 --- The team members are complete, but not appointing a person responsible for coordination  3 --- The team members are not complete, but including more than half of the people  2 --- The team members are not complete, and including less than half of the people  1 --- No emergency team |
|  |  | The hospital has emergency plan for earthquake disasters and updates it regularly.  Index illumination: The plan should include monitoring and early warning, supervision mechanism, human resources management, material management, and cooperation within and outside the hospital, and should be updated every 3 years. | 5 --- The contents of plan are complete, and updating once every 3 years or more frequently  4 --- The contents of plan are complete, but updating once more than 3 years  3 --- The contents of plan are not complete, but including more than half of the content  2 --- The contents of plan are not complete, and including less than half of the content  1 --- No plan |
|  |  | The hospital has emergency funds for disaster prevention and mitigation activities.  Index illumination: There are diversified sources of emergency funds (such as regular national financial allocations, special funds, charitable donations, international cooperation funds, etc.). | 5 --- 4 or more sources of funds  4 --- 3 sources of funds  3 --- 2 sources of funds  2 --- 1 source of fund  1 --- No fund |
|  |  | The hospital conducts earthquake disaster training or drills for medical staff regularly.  Index illumination: Contents should include triage, emergency medical treatment, personal protection, disaster management, psychological intervention, epidemic prevention, etc., and should be carried out regularly. | 5 --- The contents are complete, and conducting once every 1 years or more frequently  4 --- The contents are complete, but conducting once more than 1 years  3 --- The contents are not complete, but including more than half of the content  2 --- The contents are not complete, and including less than half of the content  1 --- No training or drill |
|  | Overload response capacity | The hospital has the capacity to augment its medical staff.  Index illumination:  ● To reserve of mobile medical personnel for disaster response.  ● Having personnel mobilization strategy, such as encourage off-duty staff to return to work, rehire retired workers.  ● Employing temporary workers with medical background, using volunteers with medical background to participate in the rescue.  ● To meet the needs of medical staff, such as the establishment of temporary care centres for staff's family members and the provision of temporary housing, food, medicine and psychological support for staff. | 5 --- 4 aspects match  4 --- 3 aspects match  3 --- 2 aspects match  2 --- 1 aspect match  1 --- No aspect match |
|  |  | The hospital is capable of expanding the number of beds.  Index illumination:  ● To plan the use of hospital beds.  ● The hospital has corresponding bed expansion measures in the case of overload, such as early discharge of stable patients, cancellation of elective surgery, using of spare beds, etc.  ● Establishing temporary treatment areas such as hospital corridors, idle rooms, etc. | 5 --- 3 aspects match  4 --- 2 aspects match  3 --- 1 aspect match  2 --- No aspect match but include few points  1 --- No aspect match |
|  |  | Most kinds of emergency medicines are available in the hospital and are checked regularly.  Index illumination: Emergency medicines mainly include antishock drugs, analgesics, anti-infective drugs, tetanus antitoxin, hemostatic drugs, trauma drugs, digestive system druges, etc. | 5 --- The kinds are complete, and checking frequently  4 --- The kinds are complete, but not checking frequently  3 --- The kinds are not complete, but including more than 2/3 drugs  2 --- The kinds are not complete, but including 1/3-2/3 drugs  1 --- The kinds are not complete, and including less than 1/3 drugs |
|  |  | The hospital has emergency supplies, or has cooperation agreements and logistics distribution strategies with material suppliers or other hospitals.  Index illumination: Emergency supplies mainly include first aid equipment (simple breathing apparatus, ecg monitors, cardiac defibrillators, oxygen cylinders, negative pressure suction devices, etc.), medical supplies (epidemic prevention and decontamination appliances, splints, bandages, personal protective appliances, etc.), power supply, clean water, food, oxygen, etc. | 5 --- The supplies are complete, or having cooperation agreements  4 --- The supplies are not complete, but including more than 2/3  3 --- The supplies are not complete, but including 1/3-2/3  2 --- The supplies are not complete, and including less than 1/3  1 --- No emergency supply |
|  |  | The hospitals is equipped with emergency communication facilities to share information in a disaster.  Index illumination: The hospital is equipped with computers, fax machines, fixed telephones, mobile phones, radio walkie-talkies and other communication facilities, which can share information with public security, fire control, emergency management and other departments. | 5 --- The communication facilities are complete, and could share information  4 --- The communication facilities are complete, but could not share information  3 --- The communication facilities are not complete, but including more than 2/3  2 --- The communication facilities are not complete, but including 1/3-2/3  1 --- The communication facilities are not complete, and including less than 1/3 |
|  | Medical staff disaster prevention and control ability | Medical staff have the ability to prepare for and mitigate earthquake disasters.  Index illumination:  ● Mastering the health status of community residents (especially the elderly, young, sick, disabled, pregnant and other vulnerable groups), have the ability to monitor and prevent diseases.  ● Having the risk prevention ability, can identify the risk that earthquake disaster may cause.  ● Having disaster education ability, can carry out earthquake disaster knowledge and skill training.  ● Having the earthquake disaster management ability, can carry on personnel and material management, participate in the formulation of disaster reduction policies. | 5 --- Extremely match  4 --- Good match  3 --- Average match  2 --- Poor match  1 --- Very poor match |
|  |  | Medical staff have the ability to assess earthquake hazards.  Index illumination:  ● Judging the damage of earthquake accurately.  ● Identifying the individuals in the high-risk environment timely and helping them evacuate quickly.  ● Familiar with the risk of the secondary disasters. | 5 --- Extremely match  4 --- Good match  3 --- Average match  2 --- Poor match  1 --- Very poor match |
|  |  | Medical staff have the ability to deal with emergency on site.  Index illumination:  ● Judging the condition of the injured correctly and implementing triage classification.  ● Having emergency rescue ability (vital signs monitoring, hemostasis, fixation, handling and transportation, cardiopulmonary resuscitation, endotracheal intubation, establishment of venous access, etc.).  ● Operating ecg monitor, defibrillator, portable ventilator, sputum aspirator and other rescue instruments skillfully.  ● Identifying the changes in the condition of the injured in time and treating effectively.  ● Having ability of managing and allocating limited resources and coordinating medical services.  ● Mastering the self-protection skills and using personal protective equipments correctly. | 5 --- Extremely match  4 --- Good match  3 --- Average match  2 --- Poor match  1 --- Very poor match |
|  |  | Medical staff have the ability to deal with post-disaster problems.  Index illumination:  ● Having ability of health education, prevention and control of infectious diseases.  ● Having ability of post-disaster psychological assessment and intervention.  ● Having ability of rehabilitation treatment/nursing.  ● Having ability of participating in post-disaster reconstruction management. | 5 --- Extremely match  4 --- Good match  3 --- Average match  2 --- Poor match  1 --- Very poor match |
|  |  | Medical staff have the ability of communication and coordination.  Index illumination: Medical staff have good communication skills and can maintain good communication and cooperation with residents, other professionals and government workers etc. | 5 --- Extremely match  4 --- Good match  3 --- Average match  2 --- Poor match  1 --- Very poor match |

**Environmental resilience**

| First-level | Second-level | Third-level | Standard score |
| --- | --- | --- | --- |
| Environmental resilience | Economic capital | Per capita disposable income.  Index illumination: Assuming that the per capita disposable income of the country in the previous year was A yuan, and the per capita disposable income of the community is B yuan. | 5 --- B>1.3A  4 --- 1.1A<B≤1.3A  3 --- 0.9A≤B≤1.1A  2 --- 0.7A≤B<0.9A  1 --- B<0.7A |
|  |  | Diversification of income structure.  Index illumination: More than 50% of households in the community have two or more sources of income (e.g., wages, rent, fund dividends, remuneration, deposit interest, investment income, etc.) that are conducive to post-disaster economic recovery. | 5 --- Above 65%  4 --- 55.01-65%  3 --- 45-55%  2 --- 35-44.99%  1 --- Under 35% |
|  |  | People who are able to work can be employed.  Index illumination: Assuming that the country unemployment rate was A, and the community unemployment rate is B. | 5 --- B<0.7A  4 --- 0.7A≤B<0.9A  3 --- 0.9A≤B≤1.1A  2 --- 1.1A<B≤1.3A  1 --- B>1.3A |
|  |  | Community property ownership rate.  Index illumination: Communities with 60% house property ownership has high disaster resilience. | 5 --- Above 78%  4 --- 66.01-78%  3 --- 54-66%  2 --- 42-53.99%  1 --- Under 42% |
|  | Disaster preparedness and response management system | Community has an earthquake disaster emergency command team.  Index illumination: Team members should include community leaders, people responsible for the environment, public security, civil affairs, publicity, family planning, property management, etc., and a appointed person responsible for coordination between different departments. | 5 --- The team members are complete, and appointing a person responsible for coordination  4 --- The team members are complete, but not appointing a person responsible for coordination  3 --- The team members are not complete, but including more than half of the people  2 --- The team members are not complete, and including less than half of the people  1 --- No emergency team |
|  |  | Community has emergency plans and policies for disaster prevention, mitigation, relief, etc.  Index illumination: Plans or policies should include supervision and management, personnel deployment, material management, departmental coordination and relief plans for vulnerable groups, and be updated every 3 years. | 5 --- The contents are complete, and updating once every 3 years or more frequently  4 --- The contents are complete, but updating once more than 3 years  3 --- The contents are not complete, but including more than half of the content  2 --- The contents are not complete, and including less than half of the content  1 --- No plans or policies |
|  |  | Communication and coordination between different functional departments.  Index illumination: Effective communication and coordination between functional departments, such as public security, public health, civil affairs, etc. | 5 --- Extremely match  4 --- Good match  3 --- Average match  2 --- Poor match  1 --- Very poor match |
|  |  | Community has disaster emergency funds for disaster prevention and mitigation activities.  Index illumination: There are diversified sources of emergency funds (such as regular national financial allocations, special funds, charitable donations, international cooperation funds, etc.). | 5 --- 4 or more sources of funds  4 --- 3 sources of funds  3 --- 2 sources of funds  2 --- 1 source of fund  1 --- No fund |
|  |  | Community has a stockpile of emergency supplies.  Index illumination: Emergency supplies mainly include lifesaving supplies (such as rescue equipments, first aid supplies, etc.) and life supplies (such as food, quilts, tents, etc.), and making full use of stores, pharmacies and warehouses in the community for storage. | 5 --- The supplies are complete, and making full use of surrounding institutions for storage  4 --- The supplies are complete, but not making full use of surrounding institutions for storage  3 --- The supplies are not complete, but including more than 1/2  2 --- The supplies are not complete, and including less than 1/2  1 --- No emergency supply |
|  |  | Community conducts earthquake emergency trainings or drills regularly.  Index illumination: Carrying out trainings or drills on risk aversion, evacuation, self and mutual rescue, post-earthquake survival and epidemic prevention regularly. | 5 --- The contents are complete, and conducting once every 1 years or more frequently  4 --- The contents are complete, but conducting once more than 1 years  3 --- The contents are not complete, but including more than 1/2  2 --- The contents are not complete, and including less than 1/2  1 --- No training or drill |
|  |  | Residents participate in community activities actively.  Index illumination: Residents can actively participate in various emergency activities and community organizations. | 5 --- Extremely match  4 --- Good match  3 --- Average match  2 --- Poor match  1 --- Very poor match |
|  |  | The number and composition of community volunteers.  Index illumination: The number of community volunteers should account for 9.75% of the total number of community residents, and the professional composition of members should be above 3 categories. | 5 --- The quantity meets the standard and the professional composition is above 6 categories  4 --- The quantity meets the standard and the professional composition is 5-6 categories  3 --- The quantity meets the standard and the professional composition is 3-4 categories  2 --- The quantity meets the standard and the professional composition is under 3 categories  1 --- The quantity does not meet the standard |
|  | Infrastructure | The water supply system is protected against earthquakes and maintained regularly.  Index illumination: The community water supply facilities are protected against earthquakes in accordance with the local seismic fortification standards, and maintained at least once a year. | 5 --- In accordance with standards and maintained once every 1/2 year  4 --- In accordance with standards and maintained once every 1/2-1 year  3 --- In accordance with standards and maintained once more than 1 year  2 --- In accordance with standards but not maintained regularly  1 --- Does not meet the standards |
|  |  | Backup water supplies can be used after the water supply is cut off.  Index illumination: There are storage tanks, stock bottled water, wells, reservoirs, artificial lakes and other landscape water; springs, rivers, lakes and other natural water as backup water sources. | 5 --- 4 or more water sources  4 --- 3 water sources  3 --- 2 water sources  2 --- 1 water source  1 --- No water source |
|  |  | The power supply system is protected against earthquakes and maintained regularly.  Index illumination: The community power supply facilities are protected against earthquakes in accordance with the local seismic fortification standards, and maintained at least once a year. | 5 --- In accordance with standards and maintained once every 1/2 year  4 --- In accordance with standards and maintained once every 1/2-1 year  3 --- In accordance with standards and maintained once more than 1 year  2 --- In accordance with standards but not maintained regularly  1 --- Does not meet the standards |
|  |  | Backup power supplies can be used if the power supply is interrupted.  Index illumination: The community should use double circuit power supplies or have uninterruptible power supplies, diesel generator sets and other emergency power supplies to ensure the power demand of important loads such as emergency lighting and fire fighting facilities. | 5 --- The community uses double circuit power supply, and critical facilities are equipped with emergency power supplies  3 --- The community uses single-circuit power, but critical facilities are equipped with emergency power supplies  1 --- The community uses single-circuit power, and critical facilities are not equipped with emergency power supplies |
|  |  | Size of the refuge meets the national standard.  Index illumination: The per capita effective refuge (park, square, stadium, etc.) should be more than 1.5m^2^ in area and the service radius should be less than 500 meters. | 5 --- Two aspects match  3 --- One aspects match  1 --- No match |
|  |  | Percentage of non-brick housing (reinforced concrete, steel frame, etc.) in the community.  Index illumination: The proportion of non-brick concrete structure housing in the community is more than 2/3, indicating that the community housing has good anti-seismic property. | 5 --- All housings are non-brick concrete structure  4 --- More than 2/3 housings are non-brick concrete structure  3 --- 1/3-2/3 housings are non-brick concrete structure  2 --- Less than 1/3 housings are non-brick concrete structure  1 --- All housings are brick concrete structure |
|  |  | Specifications of emergency roads meet national standards.  Index illumination: The main emergency roads should be at least 7 meters, and the general emergency roads should be at least 4 meters, and both of them outside of the impact of falling objects. | 5 --- Two aspects match  3 --- One aspects match  1 --- No match  (On the basis of road width score, if there is the impact of falling objects, it will be reduced by 0.5 respectively) |
|  |  | The number of roads in the community lead to the main roads outside.  Index illumination: The community should have at least 2 main roads leading to arterial roads outside. | 5 --- There are 4 or more main roads connecting with the arterial roads outside  4 --- There are 3 main roads connecting with the arterial roads outside  3 --- There are 2 main roads connecting with the arterial roads outside  2 --- There are 1 main road connecting with the arterial roads outside  1 --- There is no roads connecting with the arterial roads outside |
|  |  | The accessibility of fire rescue.  Index illumination: The fire brigade should arrive at the community within 5 minutes after the occurrence of the emergency, and the fire fighting accesses in the community should be wider than 4 meters, covering the whole community and smooth without blocking. | 5 --- The fire brigade can reach the edge of the community in 5 minutes and can run smoothly through the whole community  4 --- The fire brigade can reach the edge of the community in 5 minutes, but it can not run through the whole community only reach more than 2/3 of the community  3 --- The fire brigade can reach the edge of the community in 5 minutes, but it only reach 1/3-2/3of the community  2 --- The fire brigade can reach the edge of the community in 5 minutes, but it only reach less than 1/3of the community  1 --- The fire brigade can not reach the edge of the community in 5 minutes |
|  |  | The percentage of mobile phone usage in the community.  Index illumination: More than 80% of the residents use cell phones that can improve disaster resilience. | 5 ---92.01-100%  4 ---84.01-92%  3 ---76-84%  2 ---68-75.99%  1 ---Under 68% |
|  |  | Radio coverage in the community.  Index illumination: The coverage rate of radio stations in China is 98.9%. | 5 ---99.56-100%  4 ---99.11-99.55%  3 ---98.66-99.10%  2 ---98.23-98.67%  1 ---Under 98.23% |
