# Supplementary material for: A survey and cause analysis of community resilience in a Chinese city from the perspective of nursing
Source: BMC Public Health. 2022 Jan 15;22:2. doi: 10.1186/s12889-021-12331-1 (PMC8760825; doi:10.1186/s12889-021-12331-1)
Supplement: Supplementary file 2 — Additional file 2. Weights of three levels in the Community Resilience Evaluation System. [file 12889_2021_12331_MOESM2_ESM.docx]

**Weights of three levels in CRES-EN**

Weights of three levels in CRES-EN

| First-level |  | Second-level |  | Third-level |  |
| --- | --- | --- | --- | --- | --- |
| Content | Weight | Content | Weight | Content | Weight |
| Individual resilience | 0.343 | Health status | 0.307 | C1 I am generally in good health. | 0.331 |
|  |  |  |  | C2 I can move freely and easily. | 0.342 |
|  |  |  |  | C3 I can think clearly and communicate with others. | 0.327 |
|  |  | Mental resilience | 0.254 | C4 I believe I can control my emotions when an earthquake strikes. | 0.193 |
|  |  |  |  | C5 I always have a positive view when suffering difficulties. | 0.199 |
|  |  |  |  | C6 I do not give up easily when suffering difficulties. | 0.206 |
|  |  |  |  | C7 I can recover from setbacks quickly. | 0.212 |
|  |  |  |  | C8 I believe that difficulties make me stronger. | 0.19 |
|  |  | Social adaptation | 0.175 | C9 I can play my roles in daily life well, such as studying hard as a student, doing my own job as a worker, taking care of my family as a parent, etc. | 0.198 |
|  |  |  |  | C10 I can cope with problems well. | 0.205 |
|  |  |  |  | C11 I can adapt quickly when the environment changes. | 0.207 |
|  |  |  |  | C12 I can get along with others well. | 0.183 |
|  |  |  |  | C13 I am good at finding and using social resource (staff, funds, supplies, skills,  social relations and so on). | 0.207 |
|  |  | Disaster response capacity | 0.264 | C14 I have basic earthquake disaster assessment ability. | 0.231 |
|  |  |  |  | C15 I know how to escape or avoid danger indoors and outdoors. | 0.26 |
|  |  |  |  | C16 I have basic survival skills needed after an earthquake. | 0.26 |
|  |  |  |  | C17 I can administer first aid. | 0.249 |
| Family resilience | 0.183 | Family belief | 0.300 | C18 No matter how big the difficulties are, my families always face them bravely. | 0.511 |
|  |  |  |  | C19 We know that earthquakes are inevitable, but we believe that with the efforts  of the whole family, the damage can be minimized. | 0.489 |
|  |  | Family relationship | 0.183 | C20 The families are very united, and family members can support each other. | 0.259 |
|  |  |  |  | C21 Communication between family members is frequent and smooth. | 0.252 |
|  |  |  |  | C22 When disagreements arise between family members, we negotiate together to reach a consensus. | 0.241 |
|  |  |  |  | C23 Relationship of the families are close, and we can understand and tolerate each other. | 0.248 |
|  |  | External support | 0.248 | C24 We have close friends who can help us when we need help. | 0.244 |
|  |  |  |  | C25 We have a harmonious relationship with our neighbors and can help each other in case of an earthquake. | 0.252 |
|  |  |  |  | C26 When we are in trouble, our relatives will come to help us. | 0.256 |
|  |  |  |  | C27 Community agencies (such as neighborhood committees, police stations,  community hospitals, etc.) will help us when needed. | 0.248 |
|  |  | Crisis response | 0.269 | C28 We have an earthquake preparedness kit in our house. | 0.269 |
|  |  |  |  | C29 My family is financially sound and able to cope with life's difficulties. | 0.219 |
|  |  |  |  | C30 If an earthquake happens, we can work together to deal with the disaster. | 0.258 |
|  |  |  |  | C31 When existing methods don't work, we try to find new ways to get out of  trouble. | 0.254 |
| Health care resiience | 0.250 | Medical resource | 0.313 | C32 The ratio of the number of community health centres to the community population meets national standard. | 0.323 |
|  |  |  |  | C33 The number of health care workers per thousand population in the community. | 0.333 |
|  |  |  |  | C34 There are cooperative superior medical institutions as assistance, support or collaboration units. | 0.344 |
|  |  | Hospital emergency management | 0.361 | C35 The hospital has an earthquake emergency command team. | 0.249 |
|  |  |  |  | C36 The hospital has emergency plan for earthquake disasters and  updates it regularly. | 0.256 |
|  |  |  |  | C37 The hospital has emergency funds for disaster prevention and mitigation  Activities. | 0.235 |
|  |  |  |  | C38 The hospital conducts earthquake disaster training or drills for medical  staff regularly. | 0.26 |
|  |  | Overload response capacity | 0.150 | C39 The hospital has the capacity to augment its medical staff. | 0.187 |
|  |  |  |  | C40 The hospital is capable of expanding the number of beds. | 0.19 |
|  |  |  |  | C41 Most kinds of emergency medicines are available in the hospital and are  checked regularly. | 0.201 |
|  |  |  |  | C42 The hospital has emergency resources, or has cooperation agreements and  logistics distribution strategies with material suppliers or other hospitals. | 0.211 |
|  |  |  |  | C43 The hospital is equipped with emergency communication facilities to share  information in the event of a disaster. | 0.211 |
|  |  | Medical staff disaster prevention and control ability | 0.176 | C44 Medical staff have the ability to prepare for and mitigate earthquake disasters. | 0.203 |
|  |  |  |  | C45 Medical staff have the ability to assess earthquake hazards. | 0.196 |
|  |  |  |  | C46 Medical staff have the ability to deal with emergency on site. | 0.211 |
|  |  |  |  | C47 Medical staff have the ability to deal with post-disaster problems. | 0.196 |
|  |  |  |  | C48 Medical staff have the ability of communication and coordination. | 0.194 |
| Environmental resilience | 0.224 | Economic capital | 0.247 | C49 Per capita disposable income. | 0.258 |
|  |  |  |  | C50 Diversification of income structure. | 0.258 |
|  |  |  |  | C51 People who are able to work can be employed. | 0.249 |
|  |  |  |  | C52 Community property ownership rate. | 0.235 |
|  |  | Disaster preparedness and response management system | 0.302 | C53 Community has an earthquake disaster emergency command team. | 0.13 |
|  |  |  |  | C54 Community has emergency plans and policies for disaster prevention,  mitigation, relief, etc. | 0.132 |
|  |  |  |  | C55 Communication and coordination between different functional departments. | 0.123 |
|  |  |  |  | C56 Community has disaster emergency funds for disaster prevention and  mitigation activities. | 0.121 |
|  |  |  |  | C57 Community has a stockpile of emergency supplies. | 0.117 |
|  |  |  |  | C58 Community conducts earthquake emergency trainings or drills regularly. | 0.136 |
|  |  |  |  | C59 Residents participate in community activities actively. | 0.13 |
|  |  |  |  | C60 The number and composition of community volunteers. | 0.111 |
|  |  | Infrastructure | 0.451 | C61 The water supply system is protected against earthquakes and maintained regularly. | 0.094 |
|  |  |  |  | C62 Backup water supplies can be used after the water supply is cut off. | 0.094 |
|  |  |  |  | C63 The power supply system is protected against earthquakes and maintained  regularly. | 0.092 |
|  |  |  |  | C64 Backup power supplies can be used if the power supply is interrupted. | 0.091 |
|  |  |  |  | C65 Size of the refuge meets the national standard. | 0.095 |
|  |  |  |  | C66 Percentage of non-brick housing (reinforced concrete, steel frame, etc.) in the community. | 0.094 |
|  |  |  |  | C67 Specifications of emergency roads meet national standards. | 0.096 |
|  |  |  |  | C68 The number of roads in the community lead to the main road outside. | 0.091 |
|  |  |  |  | C69 The accessibility of fire rescue. | 0.094 |
|  |  |  |  | C70 The percentage of mobile phone usage in the community. | 0.076 |
|  |  |  |  | C71 Radio coverage in the community. | 0.083 |
